# Supplementary material for: Effect of Graphite Nanoplatelet Size and Dispersion on the Thermal and Mechanical Properties of Epoxy-Based Nanocomposites
Source: Nanomaterials (Basel). 2023 Apr 10;13(8):1328. doi: 10.3390/nano13081328 (PMC10144909; doi:10.3390/nano13081328)
Supplement: Supplementary file 1 [file nanomaterials-13-01328-s001.zip › nanomaterials-2281453-supplementary.pdf]

Supplementary information

Effect of graphite nanoplatelet size and dispersion on the thermal and mechanical properties of epoxy-based nanocomposites

Elsye Agustina, Jeungchoon Goak*, Suntae Lee, Yongse Kim, Sung Chul Hong, Yongho Seo and Naesung Lee*

Hybrid Materials Center (HMC), Department of Nanotechnology and Advanced Materials Engineering, Sejong University, 209 Neungdong-ro, Gwangjin-gu, Seoul 05006, Korea

*E-mail: jcgoak@sejong.ac.kr; nslee@sejong.ac.kr

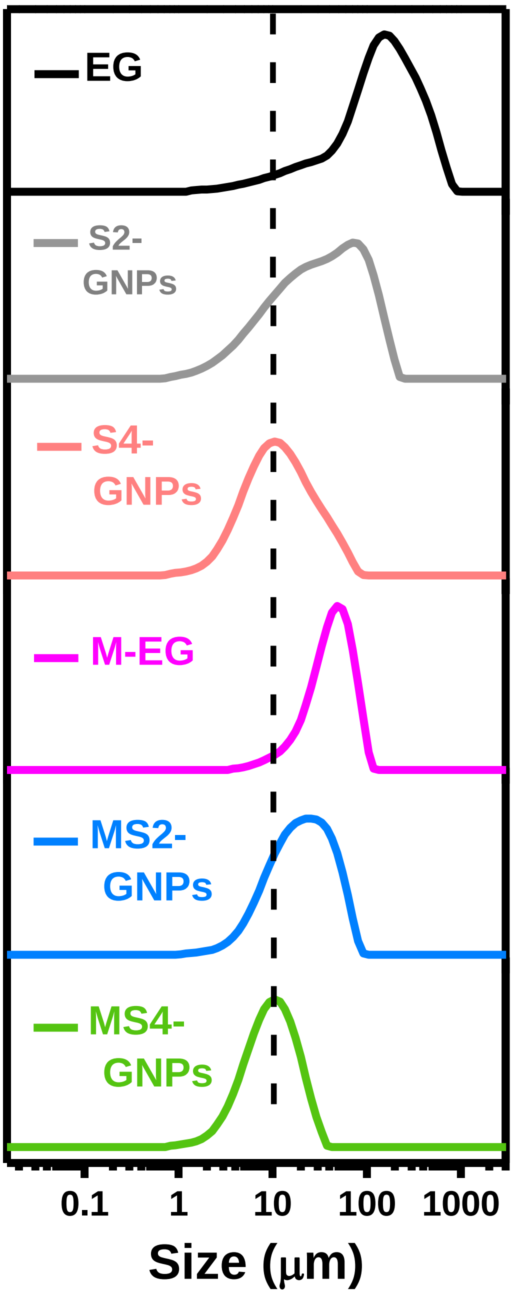

Figure S1. Particle size analyses of raw EG, S2- and S4-GNPs, M-EG, and MS2- and MS4-GNPs, measured by laser granulometry in acetone, where "S" and "M" denote sonication and milling, respectively, and the number indicates the sonication time in hours. EG: expanded graphite; GNP: graphite nanoplatelet.

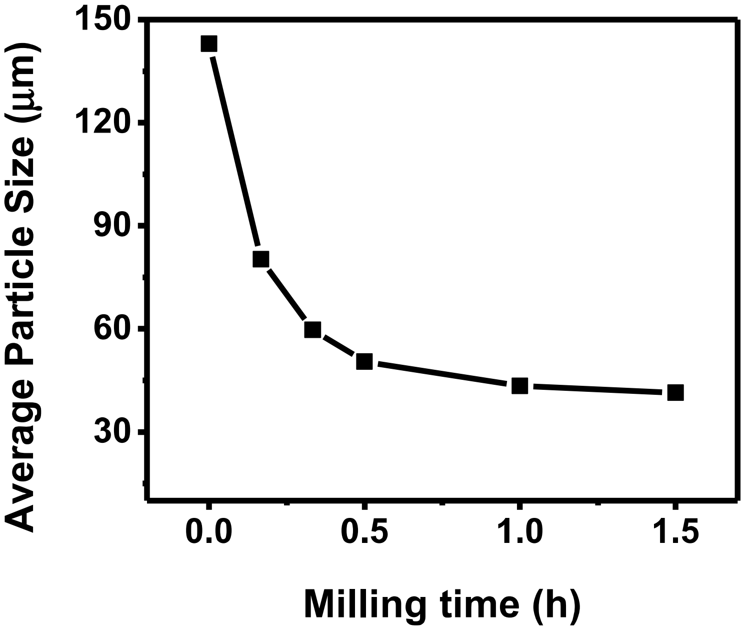

Figure S2. Average particle sizes of M-EG attrition-milled in IPA for various periods of time, measured using laser granulometry in acetone.

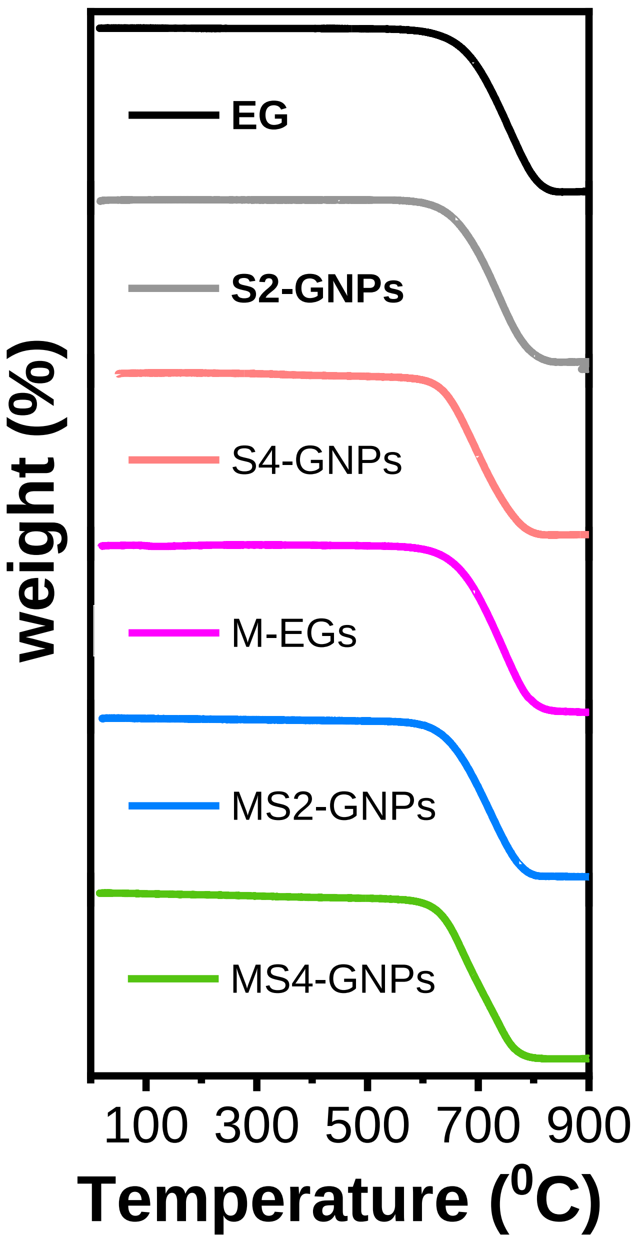

Figure S3. Thermogravimetric analysis of raw EG, S2- and S4-GNPs, M-EG, and MS2- and MS4-GNPs.

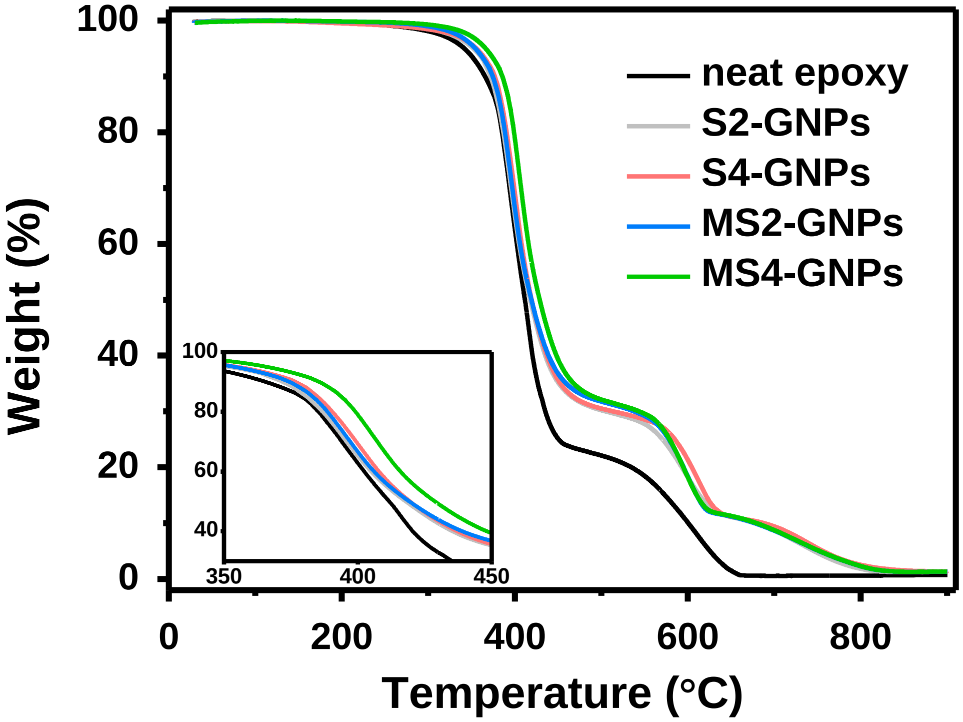

Figure S4. Thermogravimetric analysis of neat epoxy and GNP/epoxy nanocomposites with an inset to magnify the temperature region near 400°C, where epoxy decomposition occurs.

.

Table S1. Platelet sizes and thicknesses, surface areas, G-to-D peak intensity ratios (IG/ID), and oxidation temperatures (T_{ox}) of EG and GNPs, measured using SEM, AFM, BET, Raman spectroscopy, and TGA, respectively. SEM: scanning electron microscopy; AFM: atomic force microscopy; BET: Brunauer–Emmett–Teller; TGA: thermogravimetric analysis.

Samples	SEM	Laser Granulometry		BET	AFM	Raman spectroscopy	TGA
	Platelet size (μm) [a]	Particle Size (μm) [b]	FWHM (μm)	Surface area (m^2/g) [c]	Platelet thickness (nm) [d]	I_G/I_D	T_{OX} (°C)
Raw EG	-	143 ± 2.3	387.8	16.1	-	8.0 ± 3.9	743.9 ± 0.2
M-EG	-	43.0 ± 0.0	55.3	21.8	-	7.0 ± 3.2	732.4 ± 0.8
S2-GNPs	3.0 ± 2.4	31.8 ± 0.5	136.5	20.8	164. 2 ± 103.1	7.2 ± 1.2	734.4 ± 4.9
S4-GNPs	2.0 ± 1.6	11.4 ± 0.1	23.9	23.7	33.4 ± 11.3	6.2 ± 0.8	704.0 ± 3.6
MS2-GNPs	2.0 ± 1.2	20.3 ± 0.0	51.8	23.6	112.1 ± 100.1	6.3 ± 0.7	715.1 ± 1.9
MS4-GNPs	1.6 ± 0.9	9.6 ± 0.0	24.1	30.2	21.3 ± 5.8	4.9 ± 2.0	697.1 ± 1.5

[a] Platelet sizes were measured for at least 150 platelets using SEM images.

[b] Particle sizes are the average values of D_{50} sizes in the particle size distributions from three measurements.

[c] Surface areas were measured using the BET method.

[d] Platelet thicknesses were measured for at least 10 platelets using AFM.
